# Supplementary material for: Getting More Out of Biomedical Documents with GATE's Full Lifecycle Open Source Text Analytics
Source: PLoS Comput Biol. 2013 Feb 7;9(2):e1002854. doi: 10.1371/journal.pcbi.1002854 (PMC3567135; doi:10.1371/journal.pcbi.1002854)
Supplement: Dataset S1 — GATE software. Dataset S1 bundled with this paper contains a distribution of GATE (or see http://gate.ac.uk/download/). (TGZ) [file pcbi.1002854.s001.tgz › plos-gate/plugins/Lang_Hindi/resources/gazetteer/india-today-LPNETranslist_CMU_V2_1_Cleaner.htm]

@LOCATION नगा Naga -0.1693 1
@LOCATION शिव Shiva -0.1698 2
@LOCATION कहो ना Kaho Naa -0.1863 1
@LOCATION सपोतरा Sapotara -0.1894 1
@LOCATION उम्टा Umta -0.1993 1
@LOCATION सोमैया Somaiya -0.2001 2
@LOCATION यमुना Yamuna -0.2164 2
@LOCATION कोसोवो Kosovo -0.2204 1
@LOCATION विदिशा Vidisha -0.2256 1
@LOCATION मथुरा Mathura -0.2490 1
@LOCATION सौदा Sauda -0.2590 1
@LOCATION घलोटी Ghaloti -0.2609 1
@LOCATION गुना Guna -0.2643 1
@LOCATION अयोध्या Ayodhya -0.2661 5
@LOCATION डोडा Doda -0.2792 6
@LOCATION मिश्र Mishra -0.2812 4
@LOCATION टोक्यो Tokyo -0.2910 1
@LOCATION अग्निहोत्री Agnihotri -0.2927 4
@LOCATION सिमी SIMI -0.3235 2
@LOCATION जम्मू Jammu -0.3256 1
@LOCATION पुणे Pune -0.3321 2
@LOCATION ब्रह्मगिरि Brahmagiri -0.3329 1
@LOCATION जया Jaya -0.3443 3
@LOCATION मदुरै Madurai -0.3536 1
@LOCATION दुबई Dubai -0.3582 3
@LOCATION केन्या Kenya -0.3653 1
@LOCATION सेबी SEBI -0.4004 1
@LOCATION अमेरिका America -0.4407 2
@LOCATION गेक्को Gekko -0.4487 2
@LOCATION धुले Dhule -0.4596 1
@LOCATION करौली Karauli -0.4667 4
@LOCATION ब्रुनेई Brunei -0.4685 1
@LOCATION केरल Kerala -0.4864 6
@LOCATION पुरी Puri -0.5305 1
@LOCATION जेनेवा Geneva -0.5559 3
@LOCATION मध्य प्रदेश Madhya Pradesh -0.5915 10
@LOCATION कर्नाटक Karnataka -0.6954 3
@LOCATION जयललिता Jayalalitha -0.7056 21
@LOCATION दतिया Datia -0.7145 2
@LOCATION बिलमाला Bilamala -0.7222 1
@LOCATION करुणानिधि Karunanidhi -0.7322 15
@LOCATION मणिपुर Manipur -0.7737 6
@LOCATION बैलेनो Baleno -0.7978 2
@LOCATION प्रिटोरिया Pretoria -0.8115 1
@LOCATION बेटनोटी Betnoti -0.8393 2
@LOCATION रायगडा Rayagada -0.8515 2
@LOCATION मारुति Maruti -0.8810 1
@LOCATION मुरसोलि मारन Murasoli Maran -0.8838 1
@LOCATION फूलन देवी Phoolan Devi -0.9056 1
@LOCATION मनाली Manali -0.9075 1
@LOCATION चीन China -0.9442 3
@LOCATION पटना Patna -0.9468 3
@LOCATION भट्टाचार्य Bhattacharya -0.9554 17
@LOCATION गदर Gadar -0.9938 10
@LOCATION सुराणा Surana -0.9939 1
@LOCATION जिन्ना Jinnah -1.0004 5
@LOCATION कनाडा Canada -1.0287 7
@LOCATION सोलन Solan -1.0311 1
@LOCATION पानसागुडा Panasaguda -1.0506 1
@LOCATION निष्कल्प Niskalp -1.0540 7
@LOCATION लुधियाना Ludhiana -1.0719 1
@LOCATION उज्जैन Ujjain -1.0796 1
@LOCATION परना Parna -1.0829 1
@LOCATION गोद Goa -1.0885 1
@LOCATION उत्तर प्रदेश Uttar Pradesh -1.1096 11
@LOCATION नगरोटा Nagrota -1.1339 1
@LOCATION कलोल Kalol -1.1407 1
@LOCATION हमामात्सु Hamamatsu -1.1525 1
@LOCATION फूलन Phoolan -1.1631 10
@LOCATION पिताजोडी Pitajodi -1.1649 1
@LOCATION 10 जनपथ 10 Janpath -1.1707 1
@LOCATION बेहमई Behmai -1.1883 1
@LOCATION मुरसोली मारन Murasoli Maran -1.1921 1
@LOCATION सुब्रह्मण्यम Subramanyam -1.2109 4
@LOCATION सखालिन Sakhalin -1.2414 1
@LOCATION सुधाकरन Sudhakaran -1.2693 2
@LOCATION भरतपुर Bharatpur -1.2712 1
@LOCATION धर्मेंद्र Dharmendra -1.2722 3
@LOCATION एवरेस्ट Everest -1.2891 5
@LOCATION कश्मीर Kashmir -1.2909 26
@LOCATION केनरा Kerala -1.2926 1
@LOCATION जैन Jain -1.3036 13
@LOCATION इटली Italy -1.3148 1
@LOCATION भट्टाचार्य Bhattacharyas -1.3319 2
@LOCATION दोआबा Doaba -1.3352 1
@LOCATION कलकत्ता Calcutta -1.3364 1
@LOCATION गुजरात Gujarat -1.3424 8
@LOCATION बाली Bali -1.3476 2
@LOCATION देओल Deol -1.3634 2
@LOCATION हरिपाद सा Haripada Shau -1.4188 1
@LOCATION गदरः Gadar -1.4236 1
@LOCATION कुरुक्षेत्र Kurukshetra -1.4282 1
@LOCATION मुरैना Morena -1.4398 1
@LOCATION शिकागो Chicago -1.4407 1
@LOCATION भोपाल Bhopal -1.4421 2
@LOCATION धौलपुर Dhaulpur -1.4458 1
@LOCATION स्पार्क्स Sparks -1.4471 1
@LOCATION सरदार सरोवर Sardar Sarovar -1.4611 1
@LOCATION लखपत Lakhpat -1.4616 1
@LOCATION हरियाणा Haryana -1.4715 3
@LOCATION कराची Karachi -1.4777 4
@LOCATION लैक्टोजेन Lactogen -1.4782 1
@LOCATION कोरबा Korba -1.4815 1
@LOCATION रोहित बल Rohit Bal -1.4874 1
@LOCATION जालू Jalu -1.4904 2
@LOCATION पेरिस Paris -1.5000 4
@LOCATION विघ्नेश्वर गणेश Vighneswar Ganesh -1.5006 1
@LOCATION लिबर्टी Liberty -1.5051 1
@LOCATION घातली Ghatli -1.5186 1
@LOCATION मुल्तान Multan -1.5212 1
@LOCATION खालिस्तान Khalistan -1.5216 1
@LOCATION कोलकाता Kolkata -1.5382 3
@LOCATION अकासाका Akasaka -1.5386 1
@LOCATION आंध्र प्रदेश Andhra Pradesh -1.5442 3
@LOCATION मुंबई Mumbai -1.5454 33
@LOCATION डब्लिन Dublin -1.5554 1
@LOCATION कोस्टारिका , Costa Rica -1.5651 1
@LOCATION वडनगर Vadnagar -1.5744 1
@LOCATION लालू Laloo -1.5745 9
@LOCATION बिहार Bihar -1.5821 6
@LOCATION चेन्नै Chennai -1.5965 10
@LOCATION यूरोप Europe -1.6202 3
@LOCATION अरुणाचल प्रदेश Arunachal Pradesh -1.6288 3
@LOCATION नीतीश Nitish -1.6314 4
@LOCATION इंडोनेशिया Indonesia -1.6460 1
@LOCATION कोहजा Kohja -1.6480 1
@LOCATION हिमाचल प्रदेश Himachal Pradesh -1.6601 1
@LOCATION लालू यादव Laloo Yadav -1.6635 1
@LOCATION चौटाला Chautala -1.6698 9
@LOCATION तिरुवरुर Thiruvarur -1.6754 1
@LOCATION होशियारपुर Hoshiarpur -1.6802 1
@LOCATION उदयपुर Udaipur -1.7051 1
@LOCATION महाराष्ट्र Maharashtra -1.7077 8
@LOCATION रीना ढाका Rina Dhaka -1.7218 1
@LOCATION इस USA -1.7344 1
@LOCATION नेपाल Nepal -1.7373 7
@LOCATION शोर्स Shores -1.7421 1
@LOCATION लोकनायक भवन Loknayak Bhavan -1.7428 1
@LOCATION करगिल Kargil -1.7566 7
@LOCATION बच्चूभाई कास्ता Bachchubhai Kasta -1.7646 1
@LOCATION तमिलनाडु के Tamil Nadu -1.7779 4
@LOCATION राजनकुंटे Rajanakunte -1.7840 1
@LOCATION श्रीनगर Srinagar -1.7887 6
@LOCATION शेषनाग Sheshnag -1.7968 1
@LOCATION उपहार Uphaar -1.8021 1
@LOCATION पाकिस्तान Pakistan -1.8068 54
@LOCATION कश्मीरी Kashmir -1.8111 1
@LOCATION दलाल स्ट्रीट Dalal Street -1.8281 1
@LOCATION बंग्लादेश Bangladesh -1.8285 2
@LOCATION ओडीसा Orissa -1.8304 6
@LOCATION राजस्थान Rajasthan -1.8479 5
@LOCATION मलेशिया Malaysia -1.8572 1
@LOCATION आऍस्ट्रेलिया Australia -1.8642 5
@LOCATION मेडिटरेनियन Mediterranean -1.8791 1
@LOCATION गांधीनगर Gandhinagar -1.8796 1
@LOCATION तमिलनाडु से Tamil Nadu -1.8796 1
@LOCATION बड़शल्ला Barshalla -1.8826 1
@LOCATION बंगलूर Bangalore -1.8918 8
@LOCATION टर्की turkey -1.8932 1
@LOCATION जयपुर Jaipur -1.9036 2
@LOCATION चीनी china -1.9104 1
@LOCATION वैजयंतीमाला बाली Vyjayanthimala Bali -1.9255 1
@LOCATION दीपेंद्र Dipendra -1.9279 1
@LOCATION परीक्षित सागर Parikshit Sagar -1.9380 1
@LOCATION जालंधर Jalandhar -1.9383 2
@LOCATION बंगलूर पैलेस Bangalore Palace -1.9447 3
@LOCATION खार Khar -1.9450 1
@LOCATION तो राज्यसभा Rajya Sabha -1.9474 1
@LOCATION भूटान Bhutan -1.9595 1
@LOCATION मुंबईः Mumbai -1.9630 1
@LOCATION पहले Lahore -1.9637 1
@LOCATION उसे राज्यसभा Rajya Sabha -1.9649 1
@LOCATION बंगलूरः Bangalore -1.9676 1
@LOCATION बर्मिंघम Birmingham -1.9755 3
@LOCATION तिब्बत Tibet -1.9820 1
@LOCATION मिर्जापुर Mirzapur -1.9991 1
@LOCATION टर्टल Turtle -1.9994 1
@LOCATION गुवाहाटी Guwahati -2.0005 1
@LOCATION बामियान Bamiyan -2.0141 2
@LOCATION बांग्ला Bangla -2.0257 1
@LOCATION और अमेरिका South America -2.0337 1
@LOCATION मोहंती Mohanty -2.0370 5
@LOCATION कोइजाम Koijam -2.0375 4
@LOCATION मालाबार हिल Malabar Hill -2.0379 1
@LOCATION लिश्टेंस्टीन Liechtenstein -2.0516 1
@LOCATION बाबरी मस्जिद Babri Masjid -2.0517 1
@LOCATION वसंउधरा राजे Vasundhara Raje -2.0674 1
@LOCATION इंडिया INDIA -2.0694 2
@LOCATION इंडिया India -2.0694 7
@LOCATION ब्रिटेन Britain -2.0833 8
@LOCATION उसे US -2.0834 2
@LOCATION बढ़ी Delhi -2.0928 1
@LOCATION पर Bali -2.0942 1
@LOCATION वे राज्यसभा Rajya Sabha -2.1000 1
@LOCATION कैरिबजेट Caribjet -2.1069 1
@LOCATION ओलिवर रोड Oliver Road -2.1095 2
@LOCATION प्रागपुर Pragpur -2.1129 3
@LOCATION आसनसोल Asansol -2.1147 1
@LOCATION उस US -2.1152 2
@LOCATION कोलंबो Colombo -2.1178 1
@LOCATION लिबर्टीर् Liberty -2.1256 1
@LOCATION पाकिस्तानी Pakistan -2.1275 4
@LOCATION कच्छ Kutch -2.1284 4
@LOCATION बांग्लादेश Bangladesh -2.1375 4
@LOCATION लाहौर Lahore -2.1398 3
@LOCATION काबुल Kabul -2.1449 3
@LOCATION एशिया Asia -2.1624 1
@LOCATION ग्रीन पार्क Green Park -2.1649 1
@LOCATION विएतनाम Vietnam -2.1866 2
@LOCATION बंगारू लक्ष्मण Bangaru Laxman -2.1901 2
@LOCATION धौलपुर Dholpur -2.1927 2
@LOCATION इज्राएल Israel -2.1959 1
@LOCATION सिटी City -2.1962 1
@LOCATION ओंतारियो Ontario -2.2080 2
@LOCATION कश्मीर' Kashmir -2.2111 1
@LOCATION तिलक मार्ग Tilak Marg -2.2202 1
@LOCATION पेशावर Peshawar -2.2238 2
@LOCATION ग्रॉसी Grossi -2.2257 5
@LOCATION मुशर्रफ Musharraf -2.2281 20
@LOCATION डेनमार्क Denmark -2.2285 1
@LOCATION डेरा Delhi -2.2295 2
@LOCATION इस्लामाबाद Islamabad -2.2563 7
@LOCATION सिंगापुर Singapore -2.2625 4
@LOCATION तोर्शा नदी Torsha river -2.2734 1
@LOCATION बांडा एकेह Banda Aceh -2.2761 1
@LOCATION मुस्लिम-जाट बूल Muslim-Jat Ludbai -2.2808 1
@LOCATION हाजीपुर Hajipur -2.2821 3
@LOCATION मध्य प्रदेश Uttar Pradesh -2.2855 1
@LOCATION जरिया India -2.2889 1
@LOCATION देने Deol -2.2954 1
@LOCATION दिल्ली Delhi -2.2981 73
@LOCATION लौटे Laloo -2.3054 1
@LOCATION लेबनान Lebanon -2.3308 1
@LOCATION जॉर्डन Jordan -2.3336 1
@LOCATION इंदिरा India -2.3536 2
@LOCATION सिलिकान वैली Silicon Valley -2.3566 1
@LOCATION , भाउसाहब हिरे मार्ग 2 Bhausaheb Hiray Marg -2.3579 1
@LOCATION युद्ध India -2.3598 2
@LOCATION अनोखा India -2.3663 1
@LOCATION डरबन Durban -2.3697 6
@LOCATION देते Deol -2.3765 1
@LOCATION सनी देओल Sunny Deol -2.3805 2
@LOCATION असम Assam -2.3813 7
@LOCATION थाइलैंड Thailand -2.3879 1
@LOCATION याकाओलंग Yakaolang -2.3980 1
@LOCATION पवई लेक Powai Lake -2.4189 1
@LOCATION देहरादून Dehradun -2.4250 2
@LOCATION स्टरलाइट Sterlite -2.4332 4
@LOCATION सकता Kolkata -2.4389 1
@LOCATION मयूरभंज Mayurbhanj -2.4453 2
@LOCATION चारबाग Charbagh -2.4454 1
@LOCATION मंउबई Mumbai -2.4651 1
@LOCATION झारखंड Jharkhand -2.4751 2
@LOCATION अहमदाबाद Ahmedabad -2.4753 6
@LOCATION बेल्लारी, Bellary -2.4758 1
@LOCATION वाली Valley -2.4911 1
@LOCATION कोलकाता Calcutta -2.5006 1
@LOCATION चल Jalu -2.5036 1
@LOCATION गोवा Goa -2.5073 2
@LOCATION कोइज़ाम Koijam -2.5200 1
@LOCATION वसंउधरा राजे Vasundara Raje -2.5203 1
@LOCATION मुलतान Multan -2.5298 1
@LOCATION जया जेटली Jaya Jaitly -2.5402 2
@LOCATION आऍस्ट्रेलिया, Australia -2.5422 1
@LOCATION सिलिकॉन वैली Silicon Valley -2.5445 1
@LOCATION करते Kolkata -2.5453 1
@LOCATION नंदूरबार Nandurbar -2.5579 1
@LOCATION हत्या India -2.5611 1
@LOCATION देखा Delhi -2.5666 1
@LOCATION उनका India -2.5672 6
@LOCATION लगा Laloo -2.5672 1
@LOCATION माहौल Mishra -2.5692 1
@LOCATION इसका India -2.5702 2
@LOCATION लेकसाइड Lakeside -2.5714 1
@LOCATION अनुदान India -2.5717 1
@LOCATION एनीमल India -2.5745 1
@LOCATION बढ़त Kolkata -2.5776 1
@LOCATION गांगुली Ganguly -2.5785 9
@LOCATION ब्रीच कैंडी Breach Candy -2.5835 1
@LOCATION भरे Bihar -2.5919 1
@LOCATION काठमांडो Kathmandu -2.5919 2
@LOCATION म्यांमार Myanmar -2.5952 3
@LOCATION रही Delhi -2.5975 1
@LOCATION गुजरात Nigeria -2.6045 1
@LOCATION इंफाल Imphal -2.6049 2
@LOCATION गोपालनगर झील Gopalnagar lake -2.6149 1
@LOCATION भिंड Bhind -2.6150 3
@LOCATION साल sal -2.6159 6
@LOCATION सरसिंग रोंगफार Sarsing Rongphar -2.6173 1
@LOCATION गांधीधाम Gandhidham -2.6352 1
@LOCATION जापान Japan -2.6399 8
@LOCATION है sal -2.6478 1
@LOCATION मैसूर Mysore -2.6569 1
@LOCATION कर्ज Kolkata -2.6680 1
@LOCATION काजीरंगा Kaziranga -2.6715 1
@LOCATION अमेरिका Nigeria -2.6829 1
@LOCATION जल्दापाड़ा Jaldapara -2.6849 1
@LOCATION हैदराबाद Hyderabad -2.6966 6
@LOCATION जिन्ना India -2.7006 1
@LOCATION ऊमायूं रोड Humayun Road -2.7045 1
@LOCATION कक्कड़ Kakkar -2.7047 2
@LOCATION एशिया India -2.7068 2
@LOCATION ऊर्जा India -2.7079 1
@LOCATION ज्यादा Kerala -2.7153 1
@LOCATION गाजियाबाद Ghaziabad -2.7320 2
@LOCATION शेयर India -2.7343 1
@LOCATION बेकनगंज Beconganj -2.7427 1
@LOCATION रावलपिंडी Rawalpindi -2.7442 1
@LOCATION विलक्षण Varanasi -2.7465 1
@LOCATION दीवाली Diwali -2.7485 1
@LOCATION मध्य यूरोप Central Europe -2.7547 1
@LOCATION कभी Delhi -2.7560 1
@LOCATION अफगानिस्तान Afghanistan -2.7579 10
@LOCATION हृदय India -2.7582 1
@LOCATION ज्ञानेंद्र Gyanendra -2.7680 8
@LOCATION ``अयोध्या Ayodhya -2.7686 1
@LOCATION जायज India -2.7712 1
@LOCATION पांडेय Pandey -2.7743 2
@LOCATION केवलादेव नेशनल पार्क Keoladeo National Park -2.7884 1@PERSON ममता Mamata -0.0598 10
@PERSON समता Samata -0.0833 1
@PERSON पश्तो Pashto -0.0946 1
@PERSON अम्मा Amma -0.0986 5
@PERSON नमिता Namita -0.1180 5
@PERSON बिडप्पा Bidappa -0.1579 2
@PERSON सोना Sona -0.1671 2
@PERSON शिव Shiva -0.1698 2
@PERSON बोम्मई Bommai -0.1773 1
@PERSON सिन्हा Sinha -0.1873 15
@PERSON शक्ति सिन्हा Shakti Sinha -0.1905 1
@PERSON गुप्ता Gupta -0.1950 2
@PERSON सोमैया Somaiya -0.2001 2
@PERSON अशोक Ashoka -0.2110 1
@PERSON बसु Basu -0.2193 1
@PERSON शिल्पा Shilpa -0.2194 3
@PERSON शक्ति Shakti -0.2255 1
@PERSON शर्मा Sharma -0.2257 8
@PERSON हद्दा Hadda -0.2274 1
@PERSON पेमा Pema -0.2281 1
@PERSON मल्होत्रा Malhotra -0.2307 2
@PERSON नेमा Nema -0.2330 1
@PERSON पेटा PETA -0.2339 3
@PERSON सक्सेना Saksena -0.2391 1
@PERSON मथुरा Mathura -0.2490 1
@PERSON शर्मिला Sharmila -0.2712 1
@PERSON बुद्ध Buddha -0.2713 2
@PERSON डोडा Doda -0.2792 6
@PERSON मिश्र Mishra -0.2812 4
@PERSON मल्लिका Mallika -0.2926 2
@PERSON अग्निहोत्री Agnihotri -0.2927 4
@PERSON सोनी Soni -0.2951 1
@PERSON मधु सप्रे Madhu Sapre -0.3014 1
@PERSON बख्तू Bakhtu -0.3073 1
@PERSON गोपी Gopi -0.3106 3
@PERSON सेठी Sethi -0.3284 1
@PERSON मेदिनी Medini -0.3470 1
@PERSON जोशी Joshi -0.3523 7
@PERSON लो Lo -0.3525 1
@PERSON बालू Baalu -0.3555 2
@PERSON शिलोत्री Shilotri -0.3557 1
@PERSON ब्रज Braja -0.3660 4
@PERSON मुद्रा Mudra -0.3668 1
@PERSON सप्रे Sapre -0.3713 2
@PERSON द्रौपदी Draupadi -0.3720 1
@PERSON नुस्ली Nusli -0.3765 1
@PERSON रवि Ravi -0.3770 2
@PERSON जोगी Jogi -0.4313 3
@PERSON गेक्को Gekko -0.4487 2
@PERSON रोहिली Rohili -0.4561 1
@PERSON करौली Karauli -0.4667 4
@PERSON केरल Kerala -0.4864 6
@PERSON रितु बेरी Ritu Beri -0.5010 1
@PERSON मोहम्मद अली Mohammad Ali -0.5627 1
@PERSON बेरी Beri -0.5639 2
@PERSON मुहम्मद अली Muhammad Ali -0.5922 1
@PERSON लेले Lele -0.5995 2
@PERSON बिष्णुपद सेठी Bishnupada Sethi -0.6580 1
@PERSON सत्यार्थी Satyarthi -0.6580 2
@PERSON मधुबाला Madhubala -0.6613 1
@PERSON नगा होहो Naga Hoho -0.6672 1
@PERSON दिनो मोरिया Dino Morea -0.6936 1
@PERSON सोनिया Sonia -0.7013 6
@PERSON जयललिता Jayalalitha -0.7056 21
@PERSON केशुभाई Keshubhai -0.7069 2
@PERSON करुणानिधि Karunanidhi -0.7322 15
@PERSON भगत Bhagat -0.7385 1
@PERSON त्रिभुवन Tribhuvan -0.7393 1
@PERSON अनिरुद्ध बनर्जी Aniruddha Banerjee -0.7411 1
@PERSON बुद्धदेव Buddhadev -0.7447 1
@PERSON खट्टर Khattar -0.7456 2
@PERSON पार्वती Parvati -0.7549 1
@PERSON मणि रत्नम Mani Ratnam -0.7623 1
@PERSON तलौलिकर Talaulicar -0.7687 2
@PERSON जयश्री Jayashree -0.7711 1
@PERSON मणिपुर Manipur -0.7737 6
@PERSON वर्मा Verma -0.7790 5
@PERSON भीष्म कुमार अग्निहोत्री Bhisma Kumar Agnihotri -0.7821 1
@PERSON सिल्विया स्तोरै Silvia Storai -0.7898 1
@PERSON ब्रजेश मिश्र Brajesh Mishra -0.7920 2
@PERSON नरूला Narula -0.7936 1
@PERSON को सी मणि Ko Si Mani -0.7965 1
@PERSON बैलेनो Baleno -0.7978 2
@PERSON अनीता Anita -0.8015 1
@PERSON नोगुची Noguchi -0.8018 1
@PERSON मोनिषा Monisha -0.8027 1
@PERSON मिठाणी Mithani -0.8071 1
@PERSON एल्टो Alto -0.8155 1
@PERSON जेसिका Jessica -0.8306 1
@PERSON मचला Machla -0.8309 1
@PERSON मडोना Madonna -0.8326 1
@PERSON दा गिद्धा da giddha -0.8338 1
@PERSON मित्तल Mittal -0.8341 1
@PERSON बेटनोटी Betnoti -0.8393 2
@PERSON सकीना Sakina -0.8503 2
@PERSON रायगडा Rayagada -0.8515 2
@PERSON मनोज लोहिया Manoj Lohiya -0.8529 1
@PERSON शालिनी Shalini -0.8544 1
@PERSON शास्त्री Shastri -0.8565 1
@PERSON कण्णन Kannan -0.8646 1
@PERSON मौत Mamta -0.8655 1
@PERSON किरीट सोमैया Kirit Somaiya -0.8705 2
@PERSON मनोहर जोशी Manohar Joshi -0.8721 1
@PERSON मरीना Marina -0.8751 1
@PERSON अब्दुल्ला Abdullah -0.8764 3
@PERSON मारुति Maruti -0.8810 1
@PERSON मुरसोलि मारन Murasoli Maran -0.8838 1
@PERSON कपूरथला Kapurthala -0.8842 1
@PERSON गणेश Ganesh -0.8859 1
@PERSON पादुकोणे Padukone -0.8859 1
@PERSON मसानी Masani -0.8918 2
@PERSON लगान Lagaan -0.8929 1
@PERSON म्हात्रे Mhatre -0.9072 1
@PERSON विजय मुखी Vijay Mukhi -0.9098 1
@PERSON भराली Bharali -0.9133 3
@PERSON तारिणी Tarini -0.9150 1
@PERSON विद्युतमा Vidyutma -0.9352 1
@PERSON गनाई Ganai -0.9411 3
@PERSON धन Diana -0.9423 1
@PERSON सिहारे Sihare -0.9467 1
@PERSON सुभाष घई Subhash Ghai -0.9468 1
@PERSON मालिनी Malini -0.9475 2
@PERSON किरण सक्सेना Kiran Saksena -0.9494 1
@PERSON दिनेश Dinesh -0.9516 1
@PERSON पाध्या Padhya -0.9545 2
@PERSON भट्टाचार्य Bhattacharya -0.9554 17
@PERSON रीता शर्मा Rita Sharma -0.9562 1
@PERSON अली मोहम्मद गनाई Ali Mohammad Ganai -0.9586 1
@PERSON रोशन Roshan -0.9586 2
@PERSON दमन Daman -0.9638 1
@PERSON शरद जोशी Sharad Joshi -0.9648 1
@PERSON बुद्धदेव भट्टाचार्य Buddhadev Bhattacharya -0.9697 1
@PERSON ब्रज बरिक Braja Barik -0.9716 1
@PERSON रमेश Ramesh -0.9756 2
@PERSON पालेजा Paleja -0.9808 3
@PERSON मुइवा Muivah -0.9825 1
@PERSON कल्याण योजना Kalyan Yojana -0.9869 1
@PERSON नरेश खट्टर Naresh Khattar -0.9913 1
@PERSON गदर Gadar -0.9938 10
@PERSON रमानी Ramani -0.9940 1
@PERSON मोहन जेना Mohan Jena -0.9997 1
@PERSON जिन्ना Jinnah -1.0004 5
@PERSON शत्रुघ्न सिन्हा Shatrughan Sinha -1.0081 2
@PERSON बासु भट्टाचार्य Basu Bhattacharya -1.0094 1
@PERSON बजरिया Bajeria -1.0139 1
@PERSON सिखनी Sikhni -1.0146 1
@PERSON अबु हसनत Abu Hasnat -1.0163 1
@PERSON दोषी Doshi -1.0177 1
@PERSON करिश्मा Karisma -1.0280 1
@PERSON महेश म्हात्रे Mahesh Mhatre -1.0289 1
@PERSON नब जागरन Naba Jagaran -1.0296 5
@PERSON छेत्री Chhetri -1.0298 1
@PERSON कश्यप Kashyap -1.0309 1
@PERSON अब्बासी Abbassi -1.0341 1
@PERSON अबू ओसामा Abu Osama -1.0410 1
@PERSON पद्मनाभन Padmanabhan -1.0417 4
@PERSON तोर्शारानी Torsharani -1.0524 1
@PERSON निष्कल्प Niskalp -1.0540 7
@PERSON अभिजित सेन Abhijit Sen -1.0664 1
@PERSON दिनेश भट्ट Dinesh Bhatt -1.0717 1
@PERSON पद्मनाभ आचार्य Padmanabha Acharya -1.0735 1
@PERSON अब्दुल गनी लोन Abdul Gani Lone -1.0779 1
@PERSON मनोज लोहिया Manoj Lohia -1.0863 2
@PERSON परेश पालेजा Paresh Paleja -1.0916 1
@PERSON गोर्डन गेक्को Gordon Gekko -1.0957 1
@PERSON राठी Rathi -1.0976 2
@PERSON रज्जन गुज्जर Rajjan Gujjar -1.0979 1
@PERSON भरत पटेल Bharat Patel -1.1012 1
@PERSON निरुपम Nirupam -1.1094 1
@PERSON केशुभाई पटेल Keshubhai Patel -1.1117 1
@PERSON कीर्ति पटेल Kirti Patel -1.1133 1
@PERSON काशी Kashi -1.1142 1
@PERSON कटनी Katni -1.1177 1
@PERSON ब्रह्मभट्ट Brahmbhatt -1.1186 1
@PERSON मुल्ला मोहम्मद उमर Mulla Mohammad Omar -1.1194 2
@PERSON मुल्ला उमर Mulla Omar -1.1277 2
@PERSON भास्करन Bhaskaran -1.1317 1
@PERSON कमल हासन Kamal Haasan -1.1334 1
@PERSON श्रुति कार्णिक Shruti Karnik -1.1342 1
@PERSON दिनेश पटेल Dinesh Patel -1.1345 1
@PERSON सुश्रुत Susruta -1.1414 1
@PERSON मस्जिद मोठ Masjid Moth -1.1430 1
@PERSON शौरी Shourie -1.1445 1
@PERSON नीहारिका Niharika -1.1445 1
@PERSON पिनारयी विजयन Pinarayi Vijayan -1.1482 1
@PERSON रितु कुमार Ritu Kumar -1.1492 2
@PERSON प्रवीण आचार्य Pravin Aacharya -1.1493 1
@PERSON मोहम्मद अली जिन्ना Mohammed Ali Jinnah -1.1498 1
@PERSON ध्रुव Dhruv -1.1586 1
@PERSON ओलिवर स्टोन Oliver Stone -1.1607 1
@PERSON फूलन Phoolan -1.1631 10
@PERSON महेश ठक्कर Mahesh Thakkar -1.1683 1
@PERSON सूरी Suri -1.1688 2
@PERSON मनीषा Maneesha -1.1866 2
@PERSON नयनार Nayanar -1.1869 1
@PERSON वारा Vara -1.1874 3
@PERSON कैलाश सत्यार्थी Kailash Satyarthi -1.1905 1
@PERSON बनर्जी Banerjee -1.1910 1
@PERSON कैलाश शर्मा Kailash Sharma -1.1916 1
@PERSON बुश Bush -1.1919 7
@PERSON मुरसोली मारन Murasoli Maran -1.1921 1
@PERSON ए.के. मित्रा A.K. Mitra -1.2041 1
@PERSON प्रकाश जोशी Prakash Joshi -1.2045 1
@PERSON सुब्रह्मण्यम Subramanyam -1.2109 4
@PERSON मुरली मनोहर जोशी Murli Manohar Joshi -1.2111 1
@PERSON प्रकाश पादुकोणे Prakash Padukone -1.2121 1
@PERSON बीखी Bikhi -1.2180 2
@PERSON शकीना अर्किस Shakina Arcis -1.2207 1
@PERSON मदन गोपाल दमानी Madan Gopal Damani -1.2316 1
@PERSON मालिनी रमानी Malini Ramani -1.2318 2
@PERSON पल्लवी जयकिशन Pallavi Jaikishan -1.2318 1
@PERSON खादी Khadi -1.2353 1
@PERSON तरुण टहिलियाणी Tarun Tahiliani -1.2359 1
@PERSON अपर्णा कपूर Aparna Kapoor -1.2390 1
@PERSON मोहम्मद बिलाल Mohammad Bilal -1.2395 1
@PERSON सुभाष ब्रह्मभट्ट Subhash Brahmbhat -1.2422 1
@PERSON शशि रंजनः SHASHI RANJAN -1.2428 1
@PERSON करुणाकरण Karunakaran -1.2447 1
@PERSON विनय पिपरसानिया Vinay Piparsania -1.2448 1
@PERSON मीसा भारती Misa Bharati -1.2451 1
@PERSON मेलोविति दामोदरन Melovithi Damodaran -1.2465 1
@PERSON तीर्थ बहादुर श्रेष्ठ Tirtha Bahadur Shreshtha -1.2473 1
@PERSON अर्जुन Arjun -1.2499 1
@PERSON सुनील मोदी Sunil Modi -1.2503 1
@PERSON संघमित्रा भराली Sanghamitra Bharali -1.2504 1
@PERSON मुतुकरुप्पन Muthukaruppan -1.2568 1
@PERSON केरन Keran -1.2604 1
@PERSON सतीश कौशिक Satish Kaushik -1.2609 1
@PERSON लक्ष्मी Lakshmi -1.2673 1
@PERSON सुधाकरन Sudhakaran -1.2693 2
@PERSON मुझे कुछ कहना है Mujhe Kuch Kehna Hai -1.2695 1
@PERSON धर्मेंद्र Dharmendra -1.2722 3
@PERSON अर्किस Arcis -1.2739 1
@PERSON सत्तार Sattar -1.2832 1
@PERSON भंते धम्मवीरियो Bhante Dhammaviryo -1.2885 1
@PERSON शेखर कपूर Shekhar Kapur -1.2900 1
@PERSON केनरा Kerala -1.2926 1
@PERSON उमर अब्दुल्ला Omar Abdullah -1.2935 1
@PERSON मेहम Meham -1.2989 1
@PERSON सलीम अली Salim Ali -1.3013 1
@PERSON नीरीश दवे Nirish Dave -1.3020 1
@PERSON रमेश नारायणन Ramesh Narayanan -1.3033 1
@PERSON जैन Jain -1.3036 13
@PERSON अंबुमणि Anbumani -1.3039 1
@PERSON हरौडा Haroda -1.3047 1
@PERSON मेधा पाटेकर Medha Patekar -1.3057 1
@PERSON दामोदरन Damodaran -1.3089 2
@PERSON हैडोस Haydos -1.3098 1
@PERSON हनुमान Hanuman -1.3099 1
@PERSON कार्येकर Karyekar -1.3106 1
@PERSON चारू हासन Charu Haasan -1.3161 1
@PERSON राजेश खन्ना Rajesh Khanna -1.3241 1
@PERSON सरत बर्काटकी Sarat Barkataky -1.3268 1
@PERSON उमा कश्यप Uma Kashyap -1.3277 1
@PERSON रंजन किशोर भट्टाचार्य Ranjan Kishore Bhattacharya -1.3278 1
@PERSON भट्टाचार्य Bhattacharyas -1.3319 2
@PERSON जैसलमेर Jaisalmer -1.3379 1
@PERSON प्रजापत Prajapat -1.3440 3
@PERSON राऊल द कुन्हा Rahul da Cunha -1.3502 1
@PERSON राणा Rana -1.3508 1
@PERSON जयश्री गोस्वामी Jayashree Goswami -1.3529 1
@PERSON चड्ढा Chaddha -1.3534 1
@PERSON प्रकाश Prakash -1.3579 1
@PERSON कैलाश Kailash -1.3581 1
@PERSON देओल Deol -1.3634 2
@PERSON महंत Mahanta -1.3653 11
@PERSON वर्साचे Versace -1.3678 1
@PERSON अजय ठक्कर Ajay Thakkar -1.3683 1
@PERSON गौतम घोष Gautam Ghose -1.3712 1
@PERSON मीना कुमारी Meena Kumari -1.3770 1
@PERSON प्रवीण तोगड्Lया Praveen Togadia -1.3812 1
@PERSON संजीव शर्मा Sanjeev Sharma -1.3876 1
@PERSON दिवासिगमणि Deivasigamani -1.3909 1
@PERSON नवीन Naveen -1.3964 6
@PERSON अरुण Arun -1.3977 7
@PERSON विजय दिवस Vijay Divas -1.4002 2
@PERSON महाराजकुमारी मीनाक्षी देवी Maharajakumari Meenakshi Devi -1.4013 1
@PERSON पर PAE -1.4102 2
@PERSON ताइज़ुन Taizun -1.4148 1
@PERSON गिल्ली Gilly -1.4185 1
@PERSON गदरः Gadar -1.4236 1
@PERSON बासुदेव सेन Basudev Sen -1.4249 1
@PERSON महेंद्र Mahendra -1.4308 2
@PERSON अनिल कपूर Anil Kapoor -1.4368 1
@PERSON भोपाल Bhopal -1.4421 2
@PERSON हिंदुजा Hinduja -1.4459 3
@PERSON केतन पारेख Ketan Parekh -1.4469 1
@PERSON गुरभजन गिल Gurbhajan Gill -1.4475 1
@PERSON नरेश त्रेहन Naresh Trehan -1.4487 1
@PERSON विवेक Vivek -1.4491 1
@PERSON उदय Uday -1.4499 1
@PERSON पट्ट Pat -1.4513 1
@PERSON अशोक सूरी Ashok Suri -1.4581 1
@PERSON सुभाष चक्रवर्ती Subhash Chakravarty -1.4615 1
@PERSON ऊआ HuA -1.4620 1
@PERSON कमल थापा Kamal Thapa -1.4712 1
@PERSON हरियाणा Haryana -1.4715 3
@PERSON हरेन पंड्या Haren Pandya -1.4728 1
@PERSON कपूरासी-कोरियानी Kapurasi-Koriani -1.4761 1
@PERSON बासमती Basmati -1.4765 3
@PERSON बीरेन पाध्या Biren Padhya -1.4793 1
@PERSON इश्तान Ishtan -1.4798 1
@PERSON प्रमोद महाजन Pramod Mahajan -1.4813 1
@PERSON गिल Gill -1.4866 1
@PERSON रोहित बल Rohit Bal -1.4874 1
@PERSON स्तालिन Stalin -1.4893 11
@PERSON जालू Jalu -1.4904 2
@PERSON नारायणन Narayanan -1.4910 5
@PERSON महापात्र Mohapatra -1.4915 1
@PERSON अनुपम वर्मा Anupam Verma -1.4929 1
@PERSON भक्तवत्सलम Bhakthavatsalam -1.5024 1
@PERSON अब्दुल सत्तार Abdul Sattar -1.5064 1
@PERSON थुनिगेलेंग मुइवा Thunigaleng Muivah -1.5064 1
@PERSON आर्मिटेज Armitage -1.5077 1
@PERSON देवकांत महंत Devakanta Mahanta -1.5088 1
@PERSON देवयानी Devyani -1.5126 1
@PERSON योगेश त्यागी Yogesh Tyagi -1.5131 1
@PERSON कमला दास Kamala Das -1.5161 1
@PERSON गीतांजलि कश्यप Gitanjali Kashyap -1.5168 1
@PERSON नर्मदा बचाओ आंदोलन Narmada Bachao Andolan -1.5215 1
@PERSON शहीद स्मारक समिति Shaheed Smarak Samiti -1.5234 1
@PERSON नेल्सन मंडेला Nelson Mandela -1.5241 1
@PERSON शांता गोखले Shanta Gokhale -1.5263 1
@PERSON पारेख Parekh -1.5273 1
@PERSON जगदीश खट्टर Jagdish Khattar -1.5283 1
@PERSON विजय कुमार Vijay Kumar -1.5292 1
@PERSON रामनन Ramanan -1.5318 2
@PERSON रेड्डी Reddy -1.5323 6
@PERSON सुरजीत भल्ला Surjeet Bhalla -1.5359 1
@PERSON मुत्तुकरुप्पन Muthukaruppan -1.5365 3
@PERSON कोलकाता Kolkata -1.5382 3
@PERSON ब्रह्मार बरिक Brahmar Barik -1.5443 1
@PERSON मुंबई Mumbai -1.5454 33
@PERSON नूरानल Nooranal -1.5477 1
@PERSON रोमाबाई Romabai -1.5484 2
@PERSON नागमणि Nagmani -1.5492 2
@PERSON बालासोर Balasore -1.5515 1
@PERSON संभू Sambhu -1.5516 1
@PERSON दल Dal -1.5520 2
@PERSON केन नोगुची Ken Noguchi -1.5560 1
@PERSON शोला और शबनम Shola Aur Shabnam -1.5563 1
@PERSON सोंधी Sondhi -1.5612 4
@PERSON वापी Vapi -1.5629 1
@PERSON सौगत रॉय Saugata Roy -1.5643 1
@PERSON डालमिया Dalmiya -1.5679 2
@PERSON रोशन खान Roshan Khan -1.5706 1
@PERSON लालू Laloo -1.5745 9
@PERSON लज्जा कामथ Lajja Kamath -1.5759 1
@PERSON रागी ragi -1.5793 1
@PERSON सुरजीत भल्ला Surjit Bhalla -1.5835 1
@PERSON शेंबगमूर्ति Shenbagamurthy -1.5885 2
@PERSON शब्बीर मनासवाला Shabbir Manasawala -1.5915 1
@PERSON बब्बर खालसा Babbar Khalsa -1.5956 1
@PERSON चेन्नै Chennai -1.5965 10
@PERSON जतिन के Jatin K -1.5967 1
@PERSON मंजुल सिन्हा Manjul Sinha -1.5995 1
@PERSON ज़ेन Zen -1.6056 2
@PERSON किरण उत्तम घोष Kiran Uttam Ghosh -1.6118 1
@PERSON शमित सागर Shamit Saggar -1.6129 1
@PERSON निषाद Nishad -1.6147 1
@PERSON कातिए Cartier -1.6210 1
@PERSON नवीन Navin -1.6218 1
@PERSON पाठक Pathak -1.6278 1
@PERSON अजित कुमार Ajit Kumar -1.6283 1
@PERSON नीतीश Nitish -1.6314 4
@PERSON अतुल कुमार Atul Kumar -1.6320 1
@PERSON समीर Samir -1.6330 1
@PERSON के E -1.6339 1
@PERSON जगदीश गुज्जर Jagdish Gujjar -1.6346 1
@PERSON गुल Gul -1.6358 1
@PERSON मोहम्मद सईद Mohammad Sayeed -1.6359 1
@PERSON नरगिस Nargis -1.6385 2
@PERSON मारन Maran -1.6412 1
@PERSON कुमार Kumar -1.6450 3
@PERSON अर्धेंदु सारंगी Ardhendu Sarangi -1.6455 1
@PERSON अरुण शौरी Arun Shourie -1.6493 1
@PERSON ओनासिस Onassis -1.6536 1
@PERSON पुकार Pukar -1.6539 1
@PERSON राधा हत्तंगड़ी Radha Hattangadi -1.6541 1
@PERSON उमर शेख Omar Sheikh -1.6562 1
@PERSON कहना Mahanta -1.6590 1
@PERSON प्रसाद Prasad -1.6594 4
@PERSON लालू प्रसाद यादव Laloo Prasad Yadav -1.6634 1
@PERSON दीपक बुधरानी Deepak Budhrani -1.6649 1
@PERSON राघवेंद्र राठौर Raghavendra Rathore -1.6662 2
@PERSON निपामाचा Nipamacha -1.6676 3
@PERSON अब्दुल गनी भट Abdul Ghani Bhat -1.6676 1
@PERSON पूजा नय्यर Puja Nayyar -1.6687 1
@PERSON चौटाला Chautala -1.6698 9
@PERSON नंदा Nanda -1.6707 1
@PERSON तरुण ताहलियाणी Tarun Tahiliani -1.6726 1
@PERSON सतबाई Satbai -1.6741 3
@PERSON बिमल गांधी Bimal Gandhi -1.6790 1
@PERSON बिजय महापात्र Bijoy Mohapatra -1.6791 1
@PERSON अशोक भान Ashok Bhan -1.6796 1
@PERSON लिली Lily -1.6799 1
@PERSON होशियारपुर Hoshiarpur -1.6802 1
@PERSON राजन भगत Rajan Bhagat -1.6808 1
@PERSON अनंत जोशी Anant Joshi -1.6840 1
@PERSON भोगीलाल मेहता Bhogilal Mehta -1.6854 1
@PERSON इंडिका Indica -1.6864 1
@PERSON बीरबल दास धलिया Birbal Dass Dhalia -1.6879 1
@PERSON सलमान रुश्दी Salman Rushdie -1.6889 1
@PERSON अतुल भटकलकर Atul Bhatkalkar -1.6897 1
@PERSON नंजुंदप्पा Nanjundappa -1.6916 1
@PERSON वीरेंद्र जैन Virendra Jain -1.6920 1
@PERSON बृजेश मिश्र Brajesh Mishra -1.6949 1
@PERSON तृणमूल Trinamool -1.6966 1
@PERSON अल्मा मेटर Alma Mater -1.7042 4
@PERSON जमीर Jamir -1.7047 1
@PERSON स्कूटर Scooters -1.7074 1
@PERSON सक्सेना Saxena -1.7096 1
@PERSON महाजन Mahajan -1.7099 3
@PERSON हिज़्बुल मुजाहिदीन Hizb-ul Mujahideen -1.7107 1
@PERSON कुशाभाऊ ठाकरे Kushabhau Thakre -1.7111 1
@PERSON बीजू जनता दल Biju Janata Dal -1.7169 1
@PERSON महावीर Mahavir -1.7184 1
@PERSON निहाल Nihal -1.7211 1
@PERSON नेस्ले Nestle -1.7221 1
@PERSON अशोक कुमार Ashok Kumar -1.7323 1
@PERSON कल्लम अंजी रेड्डी Kallam Anji Reddy -1.7348 1
@PERSON तहलका Tehelka -1.7364 3
@PERSON गोविंद गोयल Govind Goyal -1.7370 1
@PERSON कैथरीन रूस्तमजी Katherine Rustomji -1.7398 1
@PERSON दीपा मेहता Deepa Mehta -1.7405 1
@PERSON ताज समुद्र Taj Samudra -1.7416 1
@PERSON लुई जालू Louis Jalu -1.7418 1
@PERSON किरण खेर Kiron Kher -1.7419 1
@PERSON असीम दासगुप्त Asim Dasgupta -1.7473 1
@PERSON शांति करमा Shanti Karma -1.7475 1
@PERSON अरशी अमीन Arshi Amin -1.7512 1
@PERSON नीतीश कुमार Nitish Kumar -1.7562 1
@PERSON करगिल Kargil -1.7566 7
@PERSON रघुवीर छेत्री Raghubir Chhetri -1.7570 1
@PERSON गोपीनाथ मुंडे Gopinath Munde -1.7602 1
@PERSON के पीछे हैं ke peechhe hain -1.7676 1
@PERSON कुतुबशाही Qutubshahi -1.7687 1
@PERSON अजय कायन Ajay Kayan -1.7725 1
@PERSON मुहम्मद हमजा Mohammed Hamza -1.7848 1
@PERSON ओडीसा गण परिषद Orissa Gana Parishad -1.7858 1
@PERSON परवेज़ हाशमी Parvez Hashmi -1.7859 1
@PERSON बथारी Bathery -1.7867 2
@PERSON मौलवी मोहम्मद वली Maulvi Mohammad Wali -1.7881 1
@PERSON नितिन बोरवाके Nitin Borawake -1.7887 1
@PERSON तोड़ Buddha -1.7892 1
@PERSON रमेश शाह Ramesh Shah -1.7922 1
@PERSON मेधा पाटकर Medha Patkar -1.7953 1
@PERSON इंडिगो Indigo -1.7957 1
@PERSON बिक्री Beri -1.8045 1
@PERSON केनेथ ऍगस्टीन Kenneth Augustine -1.8066 1
@PERSON केशव चौहान Keshav Chauhan -1.8082 1
@PERSON सोनिया गांधी Sonia Gandhi -1.8113 7
@PERSON दिगंबर जैन मुनि निर्भयसागर Digambar Jain Muni Nirbhaysagarji -1.8184 1
@PERSON अणे मार्ग Anne Marg -1.8199 1
@PERSON जसलोक Jaslok -1.8199 1
@PERSON के. त्रिपाठी N.K. Tripathi -1.8245 1
@PERSON लिलेट दुबे Lilette Dubey -1.8273 1
@PERSON नरेंद्र कुमार अहमद Narendra Kumar Ahmed -1.8284 1
@PERSON अमीन प्रेमजी Amin Premji -1.8292 1
@PERSON रुबी नूर Ruby Noor -1.8304 1
@PERSON ओडीसा Orissa -1.8304 6
@PERSON फिलिप थॉमस Philip Thomas -1.8316 1
@PERSON कूचबिहार के डिवीजनल Cooch Behar Divisional -1.8324 1
@PERSON शीला चड्ढा Sheela Chadha -1.8361 1
@PERSON संदीप ब्रह्मभट्ट Sandip Brahmbhatt -1.8388 1
@PERSON इस Ina -1.8392 1
@PERSON कमलेश जैन Kamlesh Jain -1.8448 1
@PERSON राजस्थान Rajasthan -1.8479 5
@PERSON यासीन मलिक Yaseen Malik -1.8544 1
@PERSON सुनील गंगोपाध्याय Sunil Gangopadhyay -1.8546 1
@PERSON तोलोलिंग Tololing -1.8677 1
@PERSON रिचर्ड आर्मिटेज Richard Armitage -1.8678 2
@PERSON रवि बजाज Ravi Bajaj -1.8687 1
@PERSON टेरी Tyrie -1.8698 1
@PERSON अनिल अंबानी Anil Ambani -1.8731 1
@PERSON केतन पारीख Ketan Parekh -1.8745 2
@PERSON नेहरू Nehru -1.8775 1
@PERSON गांधीनगर Gandhinagar -1.8796 1
@PERSON राधाबिनोद कोइजाम Radhabinod Koijam -1.8797 1
@PERSON शैलेश आयंगर Shailesh Ayyangar -1.8834 1
@PERSON एक प्रेम कथा Ek Prem Katha -1.8858 1
@PERSON शशि रंजन, Shashi Ranjan -1.8871 1
@PERSON लकपा शेरपा Lakpa Sherpa -1.8919 1
@PERSON गुरुमूरित Gurumurthy -1.8926 1
@PERSON राधाकृष्ण दमानी Radhakrishna Damani -1.8938 1
@PERSON करने Kareena -1.8963 1
@PERSON उस्मान अली खान Osman Ali Khan -1.8975 1
@PERSON रवीना टंडन Raveena Tandon -1.8982 1
@PERSON अनंत कुमार Ananth Kumar -1.9009 1
@PERSON किशोर रूंगटा Kishore Rungta -1.9061 1
@PERSON टिंकू Tinku -1.9081 1
@PERSON द्रविड़ Dravid -1.9126 4
@PERSON मिलिंद सोमण Milind Soman -1.9140 1
@PERSON अब्दुल माजिद Abdul Majid -1.9148 1
@PERSON सवाई माधोपुर Sawai Madhopur -1.9152 1
@PERSON की ASI -1.9210 1
@PERSON अब्दुल्ला शाह गाज़ी दर्घ क्लिफ्टन Abdullah Shah Gazi Dargh Clifton -1.9258 1
@PERSON सलीम-जावेद Salim-Javed -1.9322 1
@PERSON अरुंधंती Arundhanti -1.9414 1
@PERSON निशात कॉलोनी Nishat Colony -1.9472 1
@PERSON शेखर तिवारी Shekar Tiwari -1.9502 1
@PERSON गौरव बक्शी-को Gaurav Bakshi -1.9532 1
@PERSON रंजन प्रसाद यादव Ranjan Prasad Yadav -1.9542 1
@PERSON चरक Charaka -1.9546 1
@PERSON गुनीत सिंगला Gunit Singla -1.9580 1
@PERSON करण जौहर Karan Johar -1.9606 1
@PERSON बेकर Baker -1.9608 1
@PERSON मुंबईः Mumbai -1.9630 1
@PERSON केंद्र Kendra -1.9653 1
@PERSON जालंधर बरिक Jalandhar Barik -1.9672 1
@PERSON शाह Shah -1.9724 2
@PERSON शरद पवार Sharad Pawar -1.9846 1
@PERSON भराली को Bharali Rs -1.9869 1
@PERSON प्रियरंजन दासमुंशी ने Priya Ranjan Dasmunshi -1.9895 1
@PERSON आयशा प्रेम Ayesha Prem -1.9906 1
@PERSON अरुंधति राय Arundhati Roy -1.9912 1
@PERSON सुरेश अरोड़ा Suresh Arora -1.9940 1
@PERSON सभी Rabri -1.9944 1
@PERSON माणिक उपाध्याय Manik Upadhyay -1.9968 1
@PERSON के अन्नानगर Anna Nagar -1.9990 1
@PERSON नागे का पार Nage Ka Par -2.0044 1
@PERSON संजय निरुपम Sanjay Nirupam -2.0088 2
@PERSON कुदरतुल्लाह जमाल Qudratullah Jamal -2.0094 1
@PERSON बांडीपोर Bandipora -2.0121 1
@PERSON मुर्तजा Murtaza -2.0134 1
@PERSON सामी दिवल्या वनवी Sami Divlya Vanvi -2.0135 1
@PERSON बामियान Bamiyan -2.0141 2
@PERSON अशोक विश्वनाथन Ashok Viswanathan -2.0154 1
@PERSON स्टेफनो ग्रॉसी Stefano Grossi -2.0209 1
@PERSON सरूप Sarup -2.0273 1
@PERSON जगदीश Jagdish -2.0292 5
@PERSON आनंद अशोक खरे Anand Ashok Khare -2.0299 1
@PERSON राजा Raja -2.0300 6
@PERSON बरिकों Barikas -2.0304 1
@PERSON बिश्वनाथ माझी Biswanath Majhi -2.0326 1
@PERSON संपत Sampath -2.0335 1
@PERSON परकाश Parkash -2.0348 1
@PERSON मलखान Malkhan -2.0357 1
@PERSON रिपुदमन सिंह Ripudaman Singh -2.0370 1
@PERSON मोहंती Mohanty -2.0370 5
@PERSON रंजन यादव Ranjan Yadav -2.0374 3
@PERSON कोइजाम Koijam -2.0375 4
@PERSON सादिया माझी Sadia Majhi -2.0383 1
@PERSON मोहन शमशेर जंग बहादुर राणा Mohan Shamsher Jang Bahadur Rana -2.0384 2
@PERSON भान Bhan -2.0396 1
@PERSON गोपालकृष्णन Gopalakrishnan -2.0437 1
@PERSON पुल्लेला गोपीचंद Pullela Gopichand -2.0447 1
@PERSON वायको Vaiko -2.0469 1
@PERSON शब्बीर शाह Shabir Shah -2.0491 1
@PERSON नरसिंह राव Narasimha Rao -2.0502 1
@PERSON मैकबेथ Macbeth -2.0507 1
@PERSON सहिदुल आलम चौधरी Sahidul Alam Choudhury -2.0536 1
@PERSON संध्या तिवारी Sandhya Tewari -2.0560 1
@PERSON देते LeT -2.0562 1
@PERSON संबलपुर Sambalpur -2.0610 1
@PERSON ब्लेयर Blairs -2.0615 1
@PERSON हिम्मत सिंह Himmat Singh -2.0615 1
@PERSON सैयद मोदी Syed Modi -2.0618 1
@PERSON जूलिया रॉबर्ट्स Julia Roberts -2.0728 1
@PERSON एस. पद्मनाभन S. Padmanabhan -2.0808 1
@PERSON क्रिस्टोफर नेलसन Christopher Nelson -2.0825 1
@PERSON यूरोसिल Urosil -2.0925 1
@PERSON बेथनी कॉन्वेंट Bethany Convent -2.0953 1
@PERSON सौन्स Soans -2.0954 1
@PERSON मान Mann -2.0967 5
@PERSON हरिंदर बवेजा Harinder Baweja -2.0984 1
@PERSON गांधी Gandhi -2.0990 2
@PERSON बिश्वनाथ Biswanath -2.1002 3
@PERSON विजय मल्होत्रा ने Vijay Kumar Malhotra -2.1040 1
@PERSON प्रणव मुखर्जी Pranab Mukherjee -2.1043 1
@PERSON राजेश Rajesh -2.1055 1
@PERSON जसबीर कौर Jasbir Kaur -2.1077 1
@PERSON गायतोंडे Gaitonde -2.1083 1
@PERSON ओलिवर रोड Oliver Road -2.1095 2
@PERSON पॉल कक्कास्सेरी Paul Kakkassery -2.1099 1
@PERSON प्रागपुर Pragpur -2.1129 3
@PERSON विव रिचर्ड्स Viv Richards -2.1141 1
@PERSON गोयनका Goenka -2.1148 1
@PERSON सैयद अहमद Sayyed Ahmed -2.1159 1
@PERSON दलबीर कौर Dalbir Kaur -2.1170 1
@PERSON प्रफुल्ल कुमार मंहत Prafulla Kumar Mahanta -2.1176 1
@PERSON प्रशांत नंदा Prasanta Nanda -2.1222 1
@PERSON प्यूरोडर्म Puroderm -2.1260 1
@PERSON बरोज़ गज़दार Baroz Gazdar -2.1264 1
@PERSON टार्जन Tarzan -2.1277 4
@PERSON कच्छ Kutch -2.1284 4
@PERSON कोमल आनंद Komal Anand -2.1303 1
@PERSON क्योंझर Keonjhar -2.1308 1
@PERSON संजय त्यागी Sanjay Tyagi -2.1347 1
@PERSON बिल क्लिंटन Bill Clinton -2.1353 1
@PERSON सरोज ओबेरॉय Saroj Oberoi -2.1357 1
@PERSON सौरव गांगुली Sourav Ganguly -2.1363 3
@PERSON निर्भय सिंह गुज्जर Nirbhay Singh Gujjar -2.1369 1
@PERSON मुगल शेरेटन Mughal Sheraton -2.1386 1
@PERSON विक्रम मल्लाह Vikram Mallah -2.1389 1
@PERSON सपन धनंजय सिंह Sapan Dhananjay Singh -2.1423 1
@PERSON सैंट्रो Santro -2.1428 1
@PERSON राजीव दुबे Rajiv Dube -2.1428 1
@PERSON शेक्सपियर Shakespeare -2.1443 2
@PERSON मुंडे Munde -2.1443 2
@PERSON नादिर खान Nadir Khan -2.1448 1
@PERSON कमल पांडेय Kamal Pandey -2.1490 1
@PERSON इंदर प्रजापत Inder Prajapat -2.1519 1
@PERSON सैयद अली जिलानी Syed Ali Geelani -2.1523 1
@PERSON कारबी Karbi -2.1613 1
@PERSON नंगरहार Nangarhar -2.1675 1
@PERSON अभिषेक बांका Abhishek Banka -2.1688 1
@PERSON जैक स्ट्रा Jack Straw -2.1706 1
@PERSON जोए मैथ्यू Joey Mathew -2.1721 1
@PERSON राजेंद्र चतुर्वेदी, Rajendra Chaturvedi -2.1722 1
@PERSON शेन वार्न Shane Warne -2.1750 1
@PERSON अनुपम सेन, Anupam Sen -2.1764 1
@PERSON पाटकर Patkar -2.1773 1
@PERSON मुगल Mughal -2.1800 1
@PERSON मस्करेहंस Mascarenhas -2.1808 6
@PERSON दलितों Dalits -2.1856 1
@PERSON टी. सुब्बीरामी रेड्डी T. Subbirami Reddy -2.1857 1
@PERSON झील के Dal Lake -2.1876 1
@PERSON रंगराजन Rangarajan -2.1876 6
@PERSON बंगारू लक्ष्मण Bangaru Laxman -2.1901 2
@PERSON रविकांत सिन्हा कहते Ravi Kant Sinha -2.1909 1
@PERSON धौलपुर Dholpur -2.1927 2
@PERSON सुमीत नायर Sumeet Nair -2.1950 1
@PERSON दिग्विजय सिंह Digvijay Singh -2.1961 3
@PERSON जालौर Jalore -2.1992 1
@PERSON मैथ्यू सैम्युएल Mathew Samuel -2.2001 1
@PERSON रामदास Ramadoss -2.2001 2
@PERSON राऊल द्रविड़ Rahul Dravid -2.2004 2
@PERSON अटल बिहारी वाजपेयी Atal Bihari Vajpayee -2.2010 14
@PERSON मनीषा वनसिंह करमा Maneesha Vansingh Karma -2.2038 1
@PERSON निज़ाम Nizam -2.2079 4
@PERSON राऊल देव Rahul Dev -2.2108 1
@PERSON विक्रम रांगणेकर Vikram Rangnekar -2.2112 1
@PERSON नवीन पटनायक Naveen Patnaik -2.2137 2
@PERSON उसे ASI -2.2172 1
@PERSON बाबूलाल मरांडी Babulal Marandi -2.2173 1
@PERSON चार्ल्स Charles -2.2177 1
@PERSON सुंदरगढ़ Sundergarh -2.2185 1
@PERSON नीना गुप्ता की ओर Neena Gupta of Saans -2.2207 1
@PERSON चंद्रिका कुमारतुंगा Chandrika Kumaratunga -2.2221 1
@PERSON जयपाल रेड्डी Jaipal Reddy -2.2250 1
@PERSON ग्रॉसी Grossi -2.2257 5
@PERSON फीसदी Pakistanis -2.2263 2
@PERSON मुशर्रफ Musharraf -2.2281 20
@PERSON बिशन सिंह बेदी Bishan Singh Bedi -2.2285 1
@PERSON लाल लालमलानी Lal Lalmalani -2.2293 1
@PERSON चरण बरिक Charan Barik -2.2316 1
@PERSON सुनीता सिंह Sunitha Singh -2.2331 1
@PERSON सतबीर Satbir -2.2333 1
@PERSON राजे Raje -2.2349 1
@PERSON चंद्रलेखा Chandralekha -2.2350 1
@PERSON बुद्धदेव Buddha -2.2351 1
@PERSON फिर Patle -2.2403 1
@PERSON टोनी ब्लेयर Tony Blair -2.2447 1
@PERSON गिलक्रिस्ट Gilchrist -2.2464 1
@PERSON इंदिरा गांधी Indira Gandhi -2.2469 4
@PERSON ओम प्रकाश चौटाला Om Prakash Chautala -2.2504 2
@PERSON फाल्गुनी पाठक Falguni Pathak -2.2522 1
@PERSON विष्णु भागवत Vishnu Bhagwat -2.2523 2
@PERSON आनंद राठी Anand Rathi -2.2533 1
@PERSON शांता कुमार Shanta Kumar -2.2538 1
@PERSON इंदिरा राजारामन Indira Rajaraman -2.2539 1
@PERSON दयानंद सहाय Dayanand Sahay -2.2558 1
@PERSON निझावन Nijhawan -2.2586 1
@PERSON बाद Bhatt -2.2625 1
@PERSON राजीव मेनन Rajeev Menon -2.2648 1
@PERSON देवी राम Devi Ram -2.2683 3
@PERSON मुतुकृष्ण वरतराजलू Muthukrishna Varatharajaloo -2.2722 1
@PERSON शीला रावल सैंडी Sheela Raval Sandy -2.2753 1
@PERSON कश्मीरियों Kashmiris -2.2785 1
@PERSON हम आपके हैं कौन Hum Aapke Hai Koun -2.2809 1
@PERSON जगदीश चौधरी Jagdish Chaudhary -2.2817 1
@PERSON हाजीपुर Hajipur -2.2821 3
@PERSON लक्ष्मण Laxman -2.2829 1
@PERSON विलासराव देशमुख Vilasrao Deshmukh -2.2880 1
@PERSON करनजीत सिंह सेठी Karanjit Singh Sethi -2.2887 1
@PERSON कृष्णन Krishna -2.2888 1
@PERSON कानपुर Kanpur -2.2896 2
@PERSON गुरविंदर Gurvinder -2.2924 1
@PERSON हैरी बवेजा Harry Baweja -2.2929 1
@PERSON देने Deol -2.2954 1
@PERSON चोकिला अय्यर Chokila Iyer -2.2964 1
@PERSON पारीख Parekh -2.2966 3
@PERSON रामचंद्र उलाका Ramachandra Ulaka -2.2977 1
@PERSON लिए Rao -2.2992 1
@PERSON गौड़ा Gowda -2.3000 1
@PERSON भुवनेश्वर Bhubaneswar -2.3035 1
@PERSON जसबीर Jasbir -2.3041 1
@PERSON लौटे Laloo -2.3054 1
@PERSON ढेंकानाल Dhenkanal -2.3055 1
@PERSON राधाकिशन मालवीय Radhakrishan Malviya -2.3060 1
@PERSON कुट्टी नायर Kutty Nair -2.3072 1
@PERSON नवेश खंडेलवाल Navesh Khandelwal -2.3100 1
@PERSON विन्टेक Wintech -2.3107 2
@PERSON निर्मल रामप्रसाद Nirmal Ramprasad -2.3110 1
@PERSON जना कृष्णमूर्ति Jana Krishnamurthy -2.3168 1
@PERSON रंजीत कपूर Rajit Kapoor -2.3193 1
@PERSON पवन कुमारः PAWAN KUMAR -2.3204 1
@PERSON अटलबिहारी वाजपेयी से Atal Bihari Vajpayee -2.3212 1
@PERSON यशवंत सिन्हा Yashwant Sinha -2.3225 6
@PERSON निर्माता Samata -2.3253 1
@PERSON शैल राणावत Shail Ranawat -2.3255 1
@PERSON उमा भारती-के Uma Bharati -2.3277 1
@PERSON किशन राव Kishan Rao -2.3288 1
@PERSON अरुंधती रॉय Arundhati Roy -2.3289 1
@PERSON विवेक साहनी Vivek Sawhney -2.3290 1
@PERSON चिदंबरम Chidambaram -2.3344 1
@PERSON बांका Banka -2.3401 2
@PERSON पटनायक Pattanaik -2.3422 1
@PERSON जैसन बेकर Jason Baker -2.3427 1
@PERSON का Kaw -2.3481 1
@PERSON रिफात ऊसैन Rifaat Hussain -2.3484 1
@PERSON ह्रितिक Hrithik -2.3564 1
@PERSON बचते Mamata -2.3586 1
@PERSON ग्लैक्सोस्मिथक्लाइन GlaxoSmithKline -2.3606 1
@PERSON प्रशांत भूषण Prashant Bhushan -2.3637 1
@PERSON माधुरी दीक्षित Madhuri Dixit -2.3658 1
@PERSON अंशु अरोड़ा सेन Anshu Arora Sen -2.3663 1
@PERSON मोहम्मद जाफर Mohammad Jaffer -2.3672 1
@PERSON कानडा सिंह Kanada Singh -2.3698 1
@PERSON मनमोहन सिंह Manmohan Singh -2.3713 2
@PERSON माजिद Majid -2.3726 2
@PERSON सुरेश फोगला Suresh Fogla -2.3740 1
@PERSON कोफी अन्नान Kofi Annan -2.3754 1
@PERSON राजन Rajan -2.3760 1
@PERSON देते Deol -2.3765 1
@PERSON फारूक अब्दुल्ला Farooq Abdullah -2.3803 1
@PERSON ली Rs -2.3891 1
@PERSON रजनी निझावन Rajni Nijhawan -2.3955 1
@PERSON अनीता सिंह Anita Singh -2.4008 1
@PERSON दिलीप रे Dilip Ray -2.4045 1
@PERSON स्टेवरोस नियारकोस Stavros Niarchos -2.4046 1
@PERSON पैंटालून Pantaloon -2.4066 1
@PERSON परमिंदर Parminder -2.4078 1
@PERSON पर Pant -2.4116 1
@PERSON बुकर Booker -2.4127 1
@PERSON डूंगरपुर Dungarpur -2.4160 1
@PERSON भूषण Bhushan -2.4173 1
@PERSON संजय कृपाल Sanjay Kirpal -2.4198 1
@PERSON डांगी Dangi -2.4221 1
@PERSON सागर Saggar -2.4242 1
@PERSON देहरादून Dehradun -2.4250 2
@PERSON -सुप्रिया बेजबरुआ Supriya Bezbaruah -2.4272 1
@PERSON बलदेव सिंह Baldev Singh -2.4305 2
@PERSON प्रशांत भूसन Prasant Bhusan -2.4308 1
@PERSON स्टरलाइट Sterlite -2.4332 4
@PERSON मूनीर मूफारिज Mounir Moufarrige -2.4332 1
@PERSON राजा त्रिभुवन King Tribhuvan -2.4345 1
@PERSON पाते PAE -2.4366 1
@PERSON सकता Kolkata -2.4389 1
@PERSON मीर गुलाम मोहम्मद Mir Ghulam Mohammed -2.4392 1
@PERSON गुरजीत Gurjit -2.4410 2
@PERSON अरुण जेटली Arun Jaitley -2.4411 1
@PERSON वझे Vaze -2.4422 2
@PERSON मयूरभंज Mayurbhanj -2.4453 2
@PERSON हरिमोहन खन्ना कहते Hari Mohan Khanna -2.4506 1
@PERSON बंगलूर Bangalorean -2.4515 1
@PERSON अरुंधती Arundha -2.4529 1
@PERSON दाऊद इब्राहिम कासकर Dawood Ibrahim Kaskar -2.4531 2
@PERSON हैदराबाद Hyderabadi -2.4572 1
@PERSON निर्मल भंग, Nirmal Bhang -2.4636 1
@PERSON मणिशंकर अय्यर कहते Mani Shankar Aiyar -2.4650 1
@PERSON मंउबई Mumbai -2.4651 1
@PERSON गुलाम रसूल Ghulam Rasool -2.4652 1
@PERSON हरबिंदर Harbinder -2.4693 2
@PERSON आऍटोरिक्शा Autorickshaw -2.4711 1
@PERSON अहमद Ahmed -2.4729 1
@PERSON काडर Cadre -2.4768 1
@PERSON चांद मेहरा Chand Mehra -2.4773 1
@PERSON रोडनी फील्ड्स Rodney Fields -2.4782 1
@PERSON पाकिस्तानियों Pakistanis -2.4822 1
@PERSON प्रीतम की Pritam Singh -2.4836 1
@PERSON दौरान Nooranal -2.4840 1
@PERSON जवाहरलाल नेहरू Jawaharlal Nehru -2.4841 3
@PERSON मीना चावला Mina Chawla -2.4866 1
@PERSON दिया PIA -2.4874 1
@PERSON क्लिंटन Clinton -2.4888 2
@PERSON चयन बोर्ड Chandu Borde -2.4956 1
@PERSON संभू देवरी Sambhu Deuri -2.4963 1
@PERSON जैकलिन कैनेडी Jacqueline Kennedy -2.4964 1
@PERSON मुलायम सिंह यादव Mulayam Singh Yadav -2.4980 1
@PERSON चल Jalu -2.5036 1
@PERSON लाख Lancer -2.5042 1
@PERSON मुलायम, Mulayam -2.5076 1
@PERSON शेख दाऊद हसन Shaikh Dawood Hassan -2.5132 2
@PERSON दीना वाडिया Dina Wadia -2.5158 1
@PERSON रैली को Ram Lila -2.5160 1
@PERSON चंद्रबाबू नायडू Chandrababu Naidu -2.5163 1
@PERSON लिबरान Libran -2.5167 1
@PERSON कोइज़ाम Koijam -2.5200 1
@PERSON पूरी Paul -2.5240 1
@PERSON वकील अहमद मुतवक्किल Wakil Ahmed Muttawakil -2.5246 1
@PERSON ब्लॉक प्रमुख अशोक घोष Bloc supremo Ashok Ghosh -2.5276 1
@PERSON जीतू वीरवानी Jitu Virwani -2.5290 1
@PERSON सियाचिन Siachen -2.5294 1
@PERSON अमर सिंह Amar Singh -2.5301 1
@PERSON परवेज़ मुशर्रफ Pervez Musharraf -2.5303 7
@PERSON आने Dang -2.5307 1
@PERSON कोंडोलीजा राइस Condoleeza Rice -2.5311 1
@PERSON शाहजहां Shahjahan -2.5342 2
@PERSON जया जेटली Jaya Jaitly -2.5402 2
@PERSON कर Karz -2.5419 1
@PERSON जैसे Nair -2.5427 1
@PERSON करते Kolkata -2.5453 1
@PERSON प्रफुल्ल पटेल Praful Patel -2.5466 1
@PERSON अमिताभ बच्चन Amitabh Bachchan -2.5482 1
@PERSON खंडायतों Khandayats -2.5509 1
@PERSON कि S -2.5525 1
@PERSON कांति बिस्वास Kanti Biswas -2.5527 1
@PERSON पर Pret -2.5562 1
@PERSON फ्लोरा Flora -2.5567 1
@PERSON सचिन तेंडुलकर Sachin Tendulkar -2.5577 4
@PERSON मालिनी अग्रवाल Malini Aggarwal -2.5632 1
@PERSON लालू यादव RANJAN YADAV -2.5648 1
@PERSON लगा Laloo -2.5672 1
@PERSON प्रीतम सिंह Pritam Singh -2.5673 1
@PERSON भाषा ओ चेतना-के Bhasha O Chetana -2.5688 1
@PERSON माहौल Mishra -2.5692 1
@PERSON हिज़्बुल मुजाहिदीन Al-Badr Mujahideen -2.5706 1
@PERSON नायक Nayek -2.5717 1
@PERSON से A -2.5760 1
@PERSON ने A -2.5760 1
@PERSON बढ़त Kolkata -2.5776 1
@PERSON गांगुली Ganguly -2.5785 9
@PERSON पूरा करने Surya Kant -2.5789 1
@PERSON चंद्रजीत यादव Chandrajit Yadav -2.5809 1
@PERSON करण सिंह दलाल Karan Singh Dalal -2.5850 1
@PERSON कार्लसन Carlson -2.5928 1
@PERSON स्वामी Swami -2.5930 1
@PERSON रीता कायराल Reeta Kayral -2.5939 1
@PERSON स्वराज कौशल Swaraj Kaushal -2.5977 1
@PERSON राजेश प्रताप सिंह Rajesh Pratap Singh -2.5990 2
@PERSON चार्ल्स डिकेंस Charles Dickens -2.6004 1
@PERSON पाञ्चचजन्य Panchajanya -2.6010 1
@PERSON चौधरी Choudhury -2.6023 7
@PERSON स्क्वॉड्रन लीडर मोहिंदर कुमार जैन Squadron Leader Mohinder Kumar Jain -2.6037 1
@PERSON कैनेडी Kennedy -2.6041 1
@PERSON इंफाल Imphal -2.6049 2
@PERSON पावला अल्बेरताज़ी, Paola Albertazi -2.6060 1
@PERSON डाबर Dabur -2.6095 1
@PERSON तिरु Thiru -2.6118 1
@PERSON ) वाजपेयी ) Vajpayee -2.6144 1
@PERSON भिंड Bhind -2.6150 3
@PERSON दिलबर सिंह Dilbar Singh -2.6167 1
@PERSON रु Rs -2.6167 12
@PERSON सुषमा स्वराज Sushma Swaraj -2.6228 1
@PERSON होने Jon -2.6230 1
@PERSON जा Ina -2.6238 1
@PERSON ब्लेयर Blair -2.6289 3
@PERSON फारुक अब्दुल्ला Farooq Abdullah -2.6311 1
@PERSON लालाराम Lalaram -2.6315 1
@PERSON ललित मान सिंह Lalit Man Singh -2.6383 1
@PERSON नारंग Narang -2.6404 1
@PERSON तैमूर Timur -2.6422 1
@PERSON सांग लाल Saang Lal -2.6439 1
@PERSON बार Bhatt -2.6459 1
@PERSON एस्टीबन विजमैन Esteban Witzemann -2.6461 1
@PERSON इंटरवेंशनल कार्डियोलॉजी Interventional Cardiology -2.6466 1
@PERSON सिंह गुज्जर Singh Gujjar -2.6488 1
@PERSON और Fora -2.6492 1
@PERSON दाऊद हसन Dawood Hassan -2.6553 1
@PERSON राजीव गांधी Rajiv Gandhi -2.6590 4
@PERSON गुलाम हसन Ghulam Hassan -2.6608 1
@PERSON के. स्टालिन M.K. Stalin -2.6609 1
@PERSON के. स्तालिन M.K. Stalin -2.6609 1
@PERSON करुणानिधि के M. Karunanidhi -2.6657 1
@PERSON पकड़ Mahanta -2.6662 1
@PERSON राजीव त्यागी Rajiv Tyagi -2.6673 1
@PERSON कर्ज Kolkata -2.6680 1
@PERSON रेहन इंजीनियर Rehaan Engineer -2.6695 1
@PERSON बांग्लादेशियों Bangladeshis -2.6770 2
@PERSON यशवंत नाडकर्णी Yeshwant Nadkarni -2.6772 1
@PERSON विश्वभूषण हरिचंदन Biswabhusan Harichandan -2.6791 1
@PERSON हरकिशन सिंह सुरजीत Harkishan Singh Surjeet -2.6813 1
@PERSON कुतुब मीनार Qutab Minar -2.6894 1
@PERSON जैकस शिराक Jacques Chirac -2.6900 1
@PERSON मीनू आर Minoo R -2.6908 1
@PERSON इंदिरा' Indira -2.6918 1
@PERSON रखने Jaswant -2.6973 1
@PERSON यह Raja -2.7012 1
@PERSON कक्कड़ Kakkar -2.7047 2
@PERSON तारा सिंह Tara Singh -2.7110 1
@PERSON गुलाम हसन वानी Ghulam Hassan Wani -2.7138 1
@PERSON ज्यादा Kerala -2.7153 1
@PERSON एस. ओबेराय M.S. Oberoi -2.7164 1
@PERSON फातिमा बीवी Fathima Beevi -2.7195 1
@PERSON नलिनीकांत मोहंती अन्य Nalini Kanta Mohanty -2.7210 1
@PERSON गए Dal -2.7220 1
@PERSON मगर Mahajan -2.7241 1
@PERSON गाजियाबाद Ghaziabad -2.7320 2
@PERSON अजीम प्रेमजी Azim Premji -2.7325 1
@PERSON एल्विन डी'सूजा Alvin D'Souza -2.7340 1
@PERSON से R -2.7353 1
@PERSON के R -2.7353 2
@PERSON पंकज Pankaj -2.7441 1
@PERSON से T -2.7463 1
@PERSON आरिफ मसूद Arif Masood -2.7476 1
@PERSON फ्लिन रेमेडियोस Flynn Remedios -2.7488 1
@PERSON शंकर प्रसाद टेकरीवाल Shakar Prasad Tekriwal -2.7517 1
@PERSON सुरिंदर एस Surinder S -2.7533 1
@PERSON से L -2.7547 1
@PERSON ने L -2.7547 1
@PERSON के L -2.7547 3
@PERSON दे L -2.7547 1
@PERSON माधवराव सिंधिया Madhavrao Scindia -2.7575 1
@PERSON अर्जुन सिंह Arjun Singh -2.7599 2
@PERSON बोफोर्स Bofors -2.7680 1
@PERSON ज्ञानेंद्र Gyanendra -2.7680 8
@PERSON है TAC -2.7714 2
@PERSON पांडेय Pandey -2.7743 2
@PERSON कंचन Kanchana -2.7827 1
@PERSON पंत Pant -2.7855 6
@PERSON जसबीर सिंह Jasbir Singh -2.7977 1
@PERSON राजेश वनवी Rajesh Vanvi -2.7996 1
@PERSON टिकट Mamata -2.8011 1
@PERSON दयाल राम Dayal Ram -2.8083 1
@PERSON जिम्मेदारी Damodaran -2.8083 1
@PERSON तंबीदुरै Thambidurai -2.8103 1
@PERSON गोपीचंद Gopichand -2.8228 2
@PERSON , हिज़्ब-उल-मुजाहिदीन Hizb-ul Mujahideen -2.8251 1
@PERSON परवेज मुशर्रफ Pervez Musharraf -2.8273 2
@PERSON गुरेज Gurez -2.8292 1
@PERSON जगजीत सिंह चौहान Jagjit Singh Chohan -2.8301 1
@PERSON चौधरी Chowdhury -2.8334 1
@PERSON बजाज Bajaj -2.8351 5
@PERSON एस्मॉन Asmon -2.8388 1
@PERSON रूस Bush -2.8434 1
@PERSON उस Rs -2.8461 1
@PERSON ऊमायूं Humayun -2.8532 1
@PERSON कराची Advani -2.8556 1
@PERSON फैसले Pendse -2.8597 1
@PERSON गुरजीत सिंह Gurjit Singh -2.8607 1
@PERSON कोलाबा आऍप्टिशियंस Colaba Opticians -2.8613 1
@PERSON गुरबीर सिंह Gurbir Singh -2.8615 1
@PERSON और Hull -2.8619 1
@PERSON पेंडसे Pendse -2.8637 2
@PERSON एस.के. चौधरी P.S.K. Choudhary -2.8650 1
@PERSON माइकल मस्करेहंस Michael Mascarenhas -2.8667 1
@PERSON जल्दी Yaadein -2.8676 1
@PERSON वाहेंगबम निपामाचा सिंह Wahengbam Nipamacha Singh -2.8726 1
@PERSON वाजपेयी Vajpayee -2.8838 48
@PERSON बेशक Orissa -2.8842 1
@PERSON रूमा पॉल Ruma Pal -2.8844 1
@PERSON राबड़ी देवी Rabri Devi -2.8881 1
@PERSON बनाते ऊए Bansi Lal -2.8911 1
@PERSON हफीज मुहम्मद तो Hafiz Muhammad Saeed -2.8931 1
@PERSON महीने जब Man Singh -2.8932 1
@PERSON परमिंदर सिंह Parminder Singh -2.8940 1
@PERSON डंकन Duncan -2.8945 1
@PERSON राजीव चोपड़ा Rajeev Chopra -2.8968 1
@PERSON उत्तर Etawah -2.8993 1
@PERSON वीरवानी Virwani -2.8995 2
@PERSON सितारा strobe -2.9001 1
@PERSON समय Sonia -2.9016 1
@PERSON श्वार्जेनेगर Schwarzenegger -2.9018 1
@PERSON अरविंद जौहरी Arvind Johri -2.9028 1
@PERSON मेग रयान Meg Ryan -2.9030 1
@PERSON सूरज बड़जात्या Sooraj Barjatya -2.9040 1
@PERSON जेटली Jaitly -2.9072 2
@PERSON राजिंदर सच्चर Rajinder Sachar -2.9096 1
@PERSON मेरी चूनर उड़-उड़ जाए ने Meri Chunar Udd Udd Jaye -2.9126 1
@PERSON राज चोपड़ा Raj Chopra -2.9134 1
@PERSON चेनी Cheney -2.9143 1
@PERSON शैली Chennai -2.9175 1
@PERSON मुताबिक Mahanta -2.9177 1
@PERSON सफल Safal -2.9196 1
@PERSON अयोध्या Togadia -2.9231 1
@PERSON टी.के. संपत T.K. Sampath -2.9286 1
@PERSON माणक अग्रवाल Manak Agarwal -2.9319 1
@PERSON की Uri -2.9325 1
@PERSON बचन सिंह आर्य Bachan Singh Arya -2.9325 1
@PERSON बढ़ा Biswas -2.9335 1
@PERSON कमला मेघवाल Kamla Meghwal -2.9352 1
@PERSON लौट रही Mamta Nagar -2.9356 1
@PERSON हरबिंदर सिंह Harbinder Singh -2.9363 2
@PERSON पेश Sethi -2.9390 1
@PERSON इमानुल हक Imanul Huq -2.9425 1
@PERSON संग्रह Taxila -2.9439 1
@PERSON ऊई ragi -2.9469 1
@PERSON अनिल अग्रवाल Anil Agarwal -2.9507 1
@PERSON जार्ज फर्नांडीस George Fernandes -2.9540 1
@PERSON कहना Advani -2.9608 2
@PERSON रूंगटा Rungta -2.9623 2
@PERSON नंदलाल रघुवंशी Nandlal Ragoowansi -2.9660 1
@PERSON देने Pant -2.9665 1
@PERSON प्रेम सिंह Prem Singh -2.9681 2
@PERSON उमेद सिंह Umed Singh -2.9689 1
@PERSON स्यू होम्स Sue Holmes -2.9691 1
@PERSON मिली Gilly -2.9716 1
@PERSON बजाज चेतक Bajaj Chetak -2.9718 1
@PERSON वार्न Warne -2.9728 1
@PERSON अरुण सिंह Arun Singh -2.9767 2
@PERSON अरुण जेटली, Arun Jaitley -2.9779 1
@PERSON नांबियार Nambiar -2.9785 2
@PERSON ने K -2.9790 1
@PERSON के K -2.9790 4
@PERSON हरमिंदर सिंह चाथा Harminder Singh Chatha -2.9799 1
@PERSON मुमताज बेन Mumtaj Ben -2.9898 1
@PERSON मटीज Matiz -2.9906 1
@PERSON करीब Kanada -2.9911 1
@PERSON के.आर. नारायणन K.R. Narayanan -2.9924 1
@PERSON कृष्णा Krishna -2.9993 1
@PERSON अय्यर Iyer -3.0046 4
@PERSON आडवाणी Advani -3.1311 4
@PERSON निर्माता Nipamacha -3.1858 3
@PERSON बाल ठाकरे Bal Thackeray -3.1991 3
@PERSON फर्नांडीस Fernandes -3.2565 16
@PERSON चठे सेखवां Chathe Sekhwan -3.3350 3
@PERSON ठाकरे Thackeray -3.3443 4
@PERSON जसवंत Jaswant -3.4076 6
